# Krüppel-like factors in tumors: Key regulators and therapeutic avenues

**DOI:** 10.3389/fonc.2023.1080720

**Published:** 2023-01-25

**Authors:** Yuchen Zhang, Chongjie Yao, Ziyong Ju, Danli Jiao, Dan Hu, Li Qi, Shimin Liu, Xueqing Wu, Chen Zhao

**Affiliations:** ^1^ School of Acupuncture-moxibustion and Tuina, Shanghai University of Traditional Chinese Medicine, Shanghai, China; ^2^ Shuguang Hospital, Shanghai University of Traditional Chinese Medicine, Shanghai, China; ^3^ Shanghai Research Institute of Acupuncture and Meridian, Shanghai, China

**Keywords:** Krüppel-like factor, tumor, signal transduction, molecular target, tumor microenvironment

## Abstract

Krüppel-like factors (KLFs) are a group of DNA-binding transcriptional regulators with multiple essential functions in various cellular processes, including proliferation, migration, inflammation, and angiogenesis. The aberrant expression of KLFs is often found in tumor tissues and is essential for tumor development. At the molecular level, KLFs regulate multiple signaling pathways and mediate crosstalk among them. Some KLFs may also be molecular switches for specific biological signals, driving their transition from tumor suppressors to promoters. At the histological level, the abnormal expression of KLFs is closely associated with tumor cell stemness, proliferation, apoptosis, and alterations in the tumor microenvironment. Notably, the role of each KLF in tumors varies according to tumor type and different stages of tumor development rather than being invariant. In this review, we focus on the advances in the molecular biology of KLFs, particularly the regulations of several classical signaling pathways by these factors, and the critical role of KLFs in tumor development. We also highlight their strong potential as molecular targets in tumor therapy and suggest potential directions for clinical translational research.

## 1 Introduction

Krüppel-like factors (KLFs) are a transcriptional factor family of specificity protein 1-like (SP1-like) and KLFs superfamily widely expressed in multiple tissues. Controlling the expression of various genes involved in cell-cycle arrest, proliferation, apoptosis, differentiation, neurite outgrowth, and carcinogen metabolism, KLFs are always regarded as essential regulators in a broad spectrum of biological processes (1). These factors or their abnormal expressions are closely related to developing various diseases, including tumors. The individual KLFs can be tumor suppressors or oncogenes, often with context-dependent functions depending on the tissue, tumor type, or tumor stage (**Table 1**) (2).

**Table 1:** Cancers with alterations of KLFs*.

Tumor type	Expression level	
	Decreased expression	Increased expression
**Bladder cancer**	KLF4	KLF5, KLF16
**Breast cancer**	KLF2, KLF4, KLF6, KLF8-11, KLF14, KLF15, KLF17	KLF5, KLF16
**Cervical cancer**	KLF4 (of middle and low differentiation), KLF14	KLF1, KLF4 (of high differentiation)
**Clear cell carcinoma**	KLF6	
**Colorectal cancer**	KLF2-6, KLF6-SV2, KLF8-10, KLF12-15, KLF17	KLF1, KLF4 (in spheroid cells), KLF5, KLF7, KLF16
**Cutaneous squamous cell carcinoma**	KLF4	
**Endometrial cancer**	KLF9	
**Esophagus cancer**	KLF4	KLF5
**Gastric cancer**	KLF4, KLF6, KLF17	KLF1, KLF5, KLF8, KLF16
**Glioma**	KLF6, KLF9	
**Head and neck cancer**	KLF6	KLF5 (in cell nuclei), KLF6(with higher risk of local recurrence)
**Hemangioma**		KLF7 (during progressive phase, and in infantile cancer cells)
**Hepatocellular carcinoma**	KLF2, KLF4, KLF6, KLF17 (during progressive phase)	KLF4 (with sorafenib resistance), KLF5, KLF8
**Leukemia**	KLF3, KLF5	
**Lung cancer**	KLF4-6, KLF17	KLF7, KLF16
**Lymphoma**	KLF4	
**Melanoma**	KLF10	KLF5
**Myeloma**	KLF9, KLF10	
**Oral squamous cell carcinoma**	KLF6	KLF16
**Osteosarcoma**		KLF3, KLF5, KLF8
**Ovarian cancer**	KLF4	KLF8
**Pancreatic cancer**	KLF9, KLF10 (with higher risk of distant metastasis)	KLF5
**Prostate cancer**	KLF3-13, KLF17	KLF1, KLF15, KLF16
**Retinoblastoma**	KLF2	KLF16
**Thyroid cancer**	KLF17	KLF5

KLF, Krüppel-like factor. *A more detailed version of this table with references can be found in **Supplementary File S1**.

Transcriptional regulators with a zinc finger structure, like KLFs, are classified as zinc finger motifs, one of the most common motifs in the eukaryotic cell found in proteins ranging from enzymes to transcription factors (3). The basic domain mediates their direct binding with DNA and regulates gene transcription through cooperation with other activating or repressing factors. Some well-known transcription factors, such as early growth response factors, SPs, and KLFs, all belong to the Cys2/His2 (C2H2) type of zinc fingers. More than 30 zinc finger motifs have been identified (4–6). KLFs are a transcriptional factor family of SP1-like/KLFs superfamily, and the significant difference distinguishing KLFs from SPs is the absence of a Buttonhead box CXCPXC in the former (7).

In the last decade, the KLFs family has been intensively studied for its role in various aspects of tumor progression and concerning the regulation of other signaling pathways. The continued exploration of the regulatory axes of KLFs also offers the possibility of KLFs as new targets for tumor therapy. KLFs’ function in tumors is becoming a research hotspot, with more than 1400 publications in the past five years and more than 650 in the past two years alone. Thus, the study of the functions of KLFs in human tumors is a rapidly emerging field. This review focuses on the molecular regulation mediated by KLFs and recent findings regarding their vital functions in tumors. Important advances in using KLFs as a biomarker and therapeutic targets in tumor treatment are summarized, and possible directions for KLFs in transitional clinical studies are also discussed.

## 2 KLFs, a family of transcription factors with increasing interest

### 2.1 The nomenclature and classification of KLFs

Krüppel is a transcription factor initially discovered in Drosophila. The mammalian homolog to Krüppel was first identified in erythrocytes and became known as KLF1. KLFs are often named after their function or the enriched tissue at the time of identification. For example, KLF1 was originally called the Erythroid Krüppel-like factor, and KLF2 was initially called the Lung Krüppel-like factor (**Table 2**). However, according to the gene naming guidelines of the Human Gene Nomenclature Committee, gene group members should be designated by Arabic numerals immediately after the root symbol (8). Since then, 18 unique members of the KLF family have been identified and numbered by their order of discovery (KLF1-18). Notably, KLF18 is a prediction of bioinformatics, and there is no expression data reported for it, suggesting that it either has highly restricted expression patterns and specialized functions or could have become a pseudogene in extant placental mammals (9).

**Table 2:** Summary of KLFs and their aliases.

Gene	Aliases	Gene	Aliases
**KLF1**	Erythroid Krüppel-like factor	**KLF10**	Transforming growth factor-beta-inducible early growth response protein 1
**KLF2**	Lung Krüppel-Like factor	**KLF11**	Transforming growth factor-beta-inducible early growth response protein 2
**KLF3**	Basic Krüppel-Like factor	**KLF12**	Transcriptional repressor AP-2rep
**KLF4**	Epithelial Zinc finger protein Gut-enriched Krüppel-like factor	**KLF13**	Basic transcription element binding protein 3 RANTES factor of late activated T lymphocytes 1
**KLF5**	Basic transcription element binding protein 2 Intestinal-enriched Krüppel-like factor	**KLF14**	Basic transcription element binding protein 5
**KLF6**	Proto-oncogene BCD1 Core promoter element-binding protein Transcription factor Zf9	**KLF15**	Kidney-enriched Krüppel-like factor
**KLF7**	Ubiquitous Krüppel-like factor	**KLF16**	Transcription factor NSLP2 Basic transcription element binding protein 4 Dopamine receptor regulating factor
**KLF8**	Basic Krüppel-like factor 3 Zinc finger protein 741	**KLF17**	Zinc finger protein 393
**KLF9**	Basic transcription element binding protein 1	**KLF18**	/

KLF, Krüppel-like factor; BCD1, B-cell-derived protein 1; Zf, zinc finger factor; AP-2rep, activator protein-2 repressor; RANTES, regulated on activation normal T cell expressed and secreted; NSLP2, novel Sp1-like zinc finger transcription factor 2.

"/" means KLF18 do not have an aliases.

According to the structural and functional characteristics, the KLFs family is divided into the following three groups: KLF3, KLF8, and KLF12 belong to Group 1, which serve as transcriptional repressors through their interaction with the carboxyl-terminal binding protein. Group 2 includes KLF1, KLF2, KLF4, KLF5, KLF6, and KLF7, which primarily function as transcriptional activators by binding to acetyltransferases. Finally, Group 3 consists of KLF9, KLF10, KLF11, KLF13, KLF14, and KLF16, which are mainly described as transcriptional repressors because of their interaction with switch-independent-3 family member A (Sin3A), a common transcriptional corepressor. Currently, KLF15, KLF17, and KLF18 are not classified into any group, as little is known about them (9).

### 2.2 The structure of KLFs

KLFs contain two functional domains: DNA-binding and transcriptional regulatory domains. The DNA-binding domain of KLFs is located at the carboxyl terminus and contains three highly conserved C2H2 zinc finger structures. The transcriptional regulatory domain is located at the amino terminus and varies widely among members.

The DNA binding domain of KLFs contains three conserved C2H2 zinc finger structures consisting of 81 amino acids. Each zinc finger structure has a fixed length: zinc finger structure one and zinc finger structure two both have 23 amino acid residues, and zinc finger structure 3 has 21 amino acid residues (10). The C2H2 zinc finger in the KLFs consists of two short beta strands followed by an alpha helix. In the classical C2H2 zinc-finger domain, two conserved cysteines and histidine co-ordinate a zinc ion. The pattern of amino acid arrangement in a classical zinc finger is as follows: #-X-C-X (1–5)-C-X3-#-X5-#-X2-H-X(3-6)-[H/C], where C, H, and X correspond to cysteine, histidine, and any amino acid, respectively, and numbers indicate the number of residues separating the flanking amino acids (11). Zinc finger structures enable KLFs to recognize CACCC elements or GC-boxes in DNA sequences and to bind directly to important target gene regulatory regions, such as cell cycle proteins and platelet-derived growth factors. In the DNA binding region, the amino acid sequence similarity of the zinc finger motifs of KLFs is over 65%, and they have similar binding specificity to different promoters, resulting in competition for occupancy of these sites (12).

The amino terminus of KLFs is more diverse than the highly conserved carboxy terminus. The divergence at the N-terminus permits the binding of different coactivators, corepressors, or other cofactors, including histone-modifying enzymes, resulting in additional functional diversity (13). For example, KLF6 is a solid transcriptional activator, and KLF8 is a strong transcriptional repressor. KLF1 is a more typical example that integrates p300/CBP and SWI/SNF complex at the promoter and induces transcription initiation, while it also interacts with corepressors, including Sin3A and histone deacetylase 1 (HDAC1), thus transcriptionally repressing promoters *in vivo* (14).

## 3 KLFs-mediated molecular regulation

### 3.1 KLFs in NF-κB signaling

Nuclear factor kappa-B (NF-κB) is a family of transcription factor dimers formed by the combination of p50/p105/NF-κB1, p52/p100/NF-κB2, Rel/c-Rel, p65/RelA, and RelB, which plays a vital role in helping cells respond to external stimuli and coordinating cell growth and differentiation (15). When extracellular signals stimulate cells, NF-κB dimers are released from their inhibitor (IκB) and freely translocated into the nucleus to regulate the expression of their target genes (16). These responses are usually under the mediation of microRNAs (miRNAs) such as miR-547-3p and transcription factors (17).

**Figure 1:**
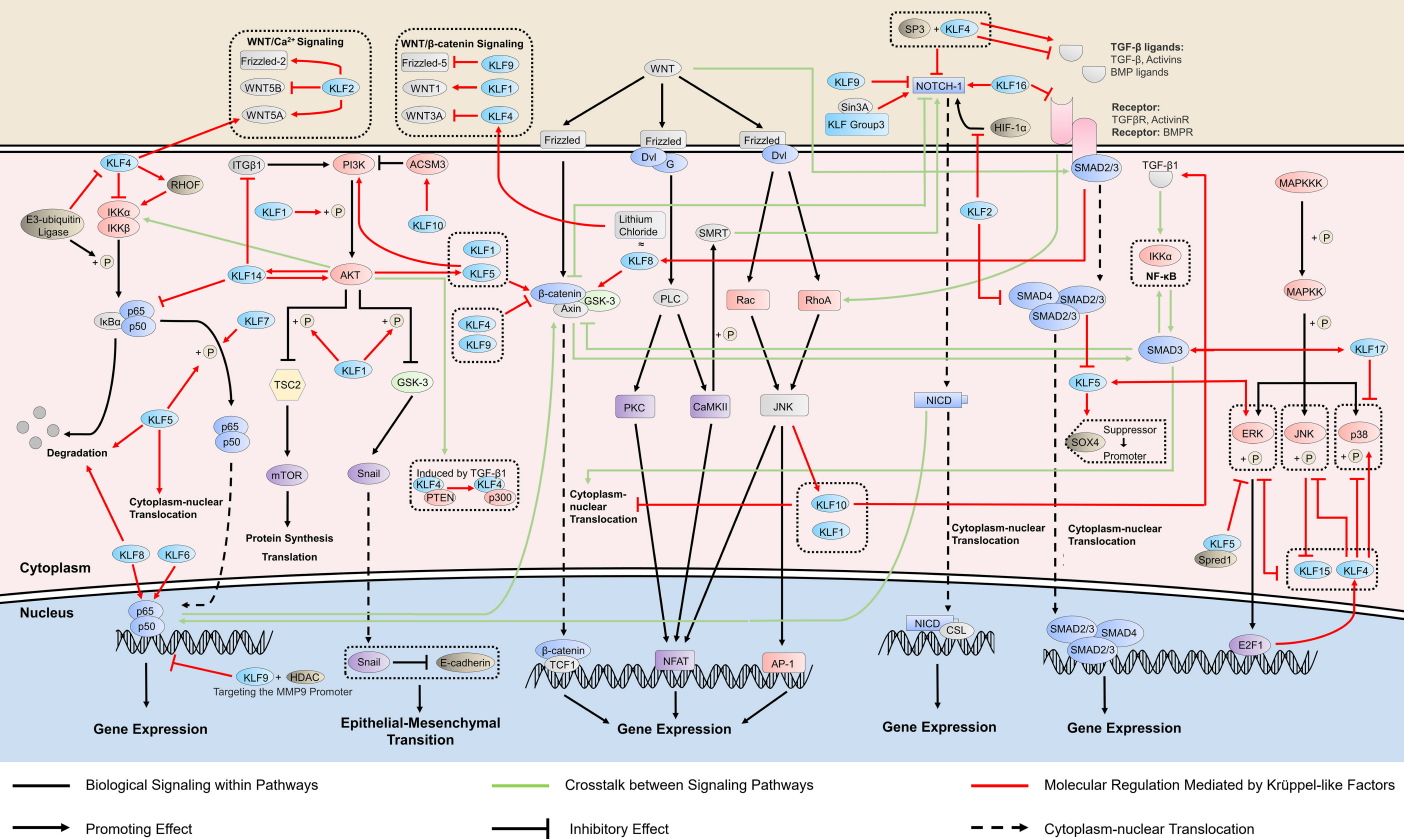
KLFs in cancer-related signaling pathways. Krüppel-like factors (KLFs) regulate multiple signaling pathways, which is crucial in cancer progression by affecting the activity of the promoter, the phosphorylation process, and the binding ability of transcription factors. The ability of some KLFs to regulate multiple signaling pathways simultaneously suggests that they may mediate the crosstalk between signaling pathways. RHO, ras homolog gene; HDAC, histone deacetylase; ITGB1, integrin subunit beta 1; PI3K, phosphatidylinositol-3-kinase; AKT, protein kinase B; TSC2, tuberous sclerosis complex 2; GSK-3, glycogen synthase kinase 3; ACSM3, acyl-CoA synthetase medium chain family member 3; PTEN, phosphatase and tensin homolog; WNT, wingless/integrated; TCF1, transcription factor 1; Dvl, dishevelled proteins; G, G protein; PLC, phospholipase C; PKC, protein kinase C; NFAT, nuclear factor of activated T cells; SMRT, silencing mediator of retinoic acid and thyroid hormone receptor; AP-1, activator protein-1; NICD, NOTCH intracellular domain; CSL, [CBF-1, Su(H), Lag-1]-type transcription factors; HIF, hypoxia-inducible factor; SOX4, sex determining region Y-box 4; TGF-β, transforming growth factor beta; MAPKKK, MAP kinase kinase kinase; MAPKK, MAP kinase kinase; ERK, extracellular regulated protein kinase; JNK, c-Jun N-terminal kinase; Spred1, Sprouty-related EVH1 domain-containing protein 1; E2F1, E2F transcription factor 1.

KLFs can physiologically regulate the NF-κB pathway (**Figure 1**). KLF2 and KLF4 are critical factors in resolution for maintaining homeostasis by mediating the negative regulatory mechanism of p300/CBP-associated factors on the inflammatory response during M1 macrophage polarization inflammation (18). Overexpression of KLF6 enhanced tumor necrosis factor-alpha (TNF-α)- and IL-1β-induced activation of NF-κB and transcription of a subset of downstream genes. Upon IL-1β stimulation, KLF6 was recruited to promoters of a subset of NF-κB target genes in a p65-dependent manner, which was, in turn, required for the optimal binding of p65 to the target gene promoters (19). KLF14 is the only KLF that can directly modulate the transcription of p65 transcription and inhibit the NF-κB signaling pathway (20).

NF-κB is a well-known signaling pathway linking inflammation and chronic diseases, including cancers, and regulates more than 500 cancer-related genes (21). KLFs also participate in cancer development by regulating NF-κB signaling (**Figure 1**). KLF4 inhibits cancer proliferation by stimulating the transforming growth factor beta 1 (TGF-β1)-mediated extracellular signal-regulated protein kinase/stress-activated protein kinase/NF-κB transcriptional program in non-small cell lung cancer (22). However, KLF4 does not only exert inhibitory effects on the NF-κB pathway. KLF4 can also induce NF-κB signaling by activating the ras homolog gene (Rho)-related GTP-binding protein RhoF in esophageal epithelia, and the KLF4-mediated chronic inflammation leads to subsequent esophageal squamous cell cancer (23). There is bidirectional regulation between KLF4 and NF-κB. TRAF7, an e3-ubiquitin ligase on the TNF-α/NF-κB pathway, can interact with the N-terminus of KLF4 protein to degrade it *via* ubiquitin, thereby eliminate the tumor-suppressor effect of KLF4 and enable cancer cell migration and invasion *in vitro* and vivo (24). KLF5, on the other hand, uses the NF-κB pathway to develop cancers such as thyroid and laryngeal carcinomas. Mechanistically, this is achieved mainly by promoting cytoplasm-nuclear translocation of NF-κB (25), or NF-κB phosphorylation at p65 and the IκBα degradation (26).

Additionally, NF-κB signaling is also regulated by other KLFs in tumor progression. KLF7 may promote the proliferation of hemangioma cells by inducing phosphorylation of p65 and IκBα, two downstream proteins in the NF-κB pathway (27). KLF8 induces breast cancer (BC) invasion through the epithelial-stromal interaction 1/valosin-containing protein/NF-κB signaling pathway, which results in the degradation of IκBα and subsequent activation of NF-κB in the nucleus (28). KLF9 inhibits BC development by interacting with HDAC1 and NF-κB p50/p65, suppressing the promoter activity of matrix metalloproteinase 9 (MMP9), an NF-κB target gene, and decreasing its expression (29). In pancreatic cancer, KLF10 may transcriptionally activate sirtuin 6 and inhibit epithelial-mesenchymal transition through NF-κB, suppressing the growth and migration ability of cancer cells (30).

NF-κB interacts with other oncogenic signaling pathways, including WNT signaling and phosphatidylinositol-3-kinase (PI3K)/protein kinase B (AKT) signaling. Elevated NF-κB signaling directly interacts with β-catenin and modulates β-catenin binding activity, which induces WNT activation and dedifferentiation of non-stem cells that acquire tumor-initiating capacity (31). PI3K/AKT/NF-κB is a crucial pathway in prostate cancer (PC) development and progression, and the IKK complex mediates the crosstalk (32).

### 3.2 KLFs in PI3K/AKT signaling

The PI3K/AKT signaling represents one of the most critical intracellular signaling pathways controlling essential cellular functions, including cell proliferation, survival, metabolism, motility, and responses to stresses and treatments. PI3K/AKT signaling hyperactivation has been observed in nearly all solid tumors. Key components within this pathway are frequently dysregulated in various tumors (33), in which KLFs may be essential regulators (**Figure 1**).

By promoting the phosphorylation of PI3K and AKT, KLF5 acts as a tumor inducer. KLF5 enhances the phosphorylation of the mammalian target of the rapamycin (mTOR) *via* the activation of PI3K/AKT signaling, thereby inhibiting autophagy in melanoma cells and enhancing tumor cell survival (34). The activated PI3K/AKT signaling increases the expression of Snail and promotes the epithelial-mesenchymal transition (EMT) in hepatocellular carcinoma (HCC) (35). Hypoxia-mediated chemoresistance has been regarded as an important obstacle in the development of cancer treatment. KLF5 knockdown suppresses hypoxia-induced cisplatin resistance by inhibiting hypoxia-inducible factor-1-dependent glycolysis *via* the inactivation of the PI3K/AKT/mTOR pathway (36). Interestingly, some experiments propose that PI3K signaling increases the expression of KLF5 by inhibiting miR-9, which targets KLF5, indicating a potential positive feedback regulation between KLF5 and PI3K/AKT signaling (37).

KLF14 is also closely related to PI3K/AKT signaling. KLF14 activates this signaling by enhancing the activation of AKT kinase and preventing AKT phosphorylation (38). Nevertheless, KLF14 simultaneously suppresses cervical cancer progression by inhibiting integrin beta 1 transcription, which upregulates key molecules in the PI3K/AKT signaling, including AKT and p-AKT, representing an indirect inhibitory effect of KLF14 on PI3K/AKT (39). The interaction between KLF14 and PI3K is also bidirectional. In PC, KLF14 is a critical factor in regulating antioxidant response and subsequent pathogenesis of castration resistance. Its expression decreased after intervention with inhibitors of the PI3K/AKT pathway, suggesting its upregulation partly depends on the PI3K/AKT pathway (40).

Several other KLFs also participate in the regulation of PI3K/AKT signaling. KLF1 promotes metastasis and invasion *via* the PI3K/AKT signaling pathway in cervical cancer cells by enhancing the phosphorylation levels of PI3K and AKT (41). KLF10 inhibits melanoma cells’ proliferation, invasion, and migration by upregulating acyl−CoA medium−chain synthetase 3. This regulation is associated with inhibiting phosphorylation levels of PI3K and AKT by KLF10 (42).

PI3K/AKT mediates the TGF-β1-induced alteration of KLF4 acetylation status and subsequent histone H3 acetylation. After TGF-β1 signaling activation, phosphatase and tensin homolog (PTEN) was phosphorylated by p38 MAPK or PI3K/AKT signaling. The phosphorylated PTEN lost its ability to dephosphorylate KLF4, and the cofactors interacting with KLF4 switched from PTEN to p300. Then, KLF4-p300 complexes were recruited to KLF4-binding sites of vascular smooth muscle cells gene promoter to acetylate histone H3 and activate transcription (43). PI3K/AKT also mediates the transduction between TGF-β and NF-κB, and TGF-β/PI3K/AKT/mTOR/NF-κB transduction pathway may bring new possibilities for predicting the prognosis and early diagnosis of cancer (44).

### 3.3 KLFs in WNT signaling

Wingless/integrated proteins (WNTs) are secreted glycoproteins comprising 19 members that can bind to cell surface receptors, including Frizzled (FZD) family receptors and co-receptors such as receptor tyrosine kinase-like orphan receptors (ROR1/2), and receptor-like tyrosine kinase (45). The WNT signaling pathway is a critical regulator of development and adult tissue homeostasis and becomes dysregulated in many tumor types (46). Classically, the canonical WNT signaling activation stabilizes cytoplasmic β-catenin which subsequently translocate into the nucleus for the transcription of WNT-specific genes, while activating the non-canonical WNT pathway does not use β-catenin (47).

KLF4, KLF9, and KLF10 have antitumor effects by inhibiting the canonical WNT/β-catenin signaling pathway (**Figure 1**). KLF4 can restrain the migration and proliferation of cancer cells by inhibiting downstream molecules of WNT signaling, such as cyclinD1 and c-Myc (48), and directly regulating the WNT/β-catenin pathway to inhibit EMT (49). However, some studies (50) suggest that KLF4 can be regulated by the canonical WNT pathway activator lithium chloride, indicating that canonical WNT activation directly increases KLF4 expression and a KLF4-mediated β-catenin activation. The interaction between KLF4 and this signaling pathway has become the target of multiple microRNAs involved in tumor regulation. As a direct target of miR-7-5p, KLF4 mediates its inhibition of the proliferation and migration of esophageal cancer cells through the regulation of WNT3A and β-catenin protein (51). MiR-92a could also bind to the 3’-UTR of the KLF4 gene and relieve its inhibitory effect on WNT signaling. After miR-92a silencing, the expressions of WNT/β-catenin pathway-associated proteins were remarkably down-regulated, including β-catenin, c-Myc, and WNT3A (52). Animal experiments have demonstrated that the WNT gene is a target gene for KLF9 (53) which inhibits this signaling pathway to reduce the proliferation and invasion of cancer cells (54). Further studies suggest that the inhibitory effect of KLF9 on the WNT pathway is achieved by negatively regulating FZD-5 (55), a vital component of the WNT pathway, at both the mRNA and protein levels or/and down-regulating β-catenin activity (54). Under the regulation of miR-106b-5p, KLF10 functions as a tumor suppressor in multiple myeloma by inhibiting β-catenin nuclear translocation (56).

KLF1, KLF5, and KLF8 promote tumor proliferation by activating the expression of the WNT/β-catenin pathway (**Figure 1**). The knockdown of KLF1 in gastric cancer inhibited the growth of tumor cells and the activation of the canonical WNT signaling pathway. The decreased expression levels of WNT1, cytoplasmic and nuclear β-catenin, and downstream targets c-Myc and cyclin D1 suggest that KLF1 promotes the proliferation and EMT progression of tumor cells by activating this signaling pathway (57). KLF8 plays the exact biological role as lithium chloride that activates the canonical WNT signaling and maintains the stem cell-like characteristics of tumor cells, thus achieving strong tumorigenicity (58). KLF5 acts as a fundamental core regulator of intestinal oncogenesis at the stem-cell level, enhancing the nuclear localization and transcriptional activity of β-catenin, aiding tumor initiation (59), which is the target for a variety of antitumor drugs (60, 61).

The non-canonical WNT signaling pathways are classified into either the planar cell polarity (PCP) pathway or the WNT/Ca^2+^ pathway according to specific WNT ligands and their binding receptors and co-receptors (47), and both of them are regulated by KLFs (**Figure 1**). The overexpression of KLF2 affected WNT5A, WNT5B, and FZD-2 in the WNT signaling pathway, which regulates epithelial-mesenchymal transition involved in tumor metastasis (62). KLF4 may be involved in tumor development through the WNT5A signaling pathway, a WNT pathway activated by the receptor tyrosine kinase ROR2. WNT5A is a direct transcriptional target of KLF4 activation in squamous epithelial cells, and KLF4 can regulate CDC42, a small GTPase protein, in a WNT5A-dependent manner (63). The aberrant expression of CDC42 is strongly associated with the development of a variety of tumors (64), and thus, it may be a downstream product of KLF4 involvement in tumor progression. KLF10, also known as transforming growth factor-beta-inducible early growth response protein 1, can be induced by WNT/PCP/c-Jun amino-terminal kinase (JNK) signaling, indicating KLF10 may mediate the crosstalk between the WNT signaling and TGF-β signaling (65).

WNT signaling has extensive crosstalk with NOTCH pathways in tumor development. In canonical WNT signaling, the β-catenin interacts with NOTCH-1 and decreases its ubiquitination, causing elevated Hes-1 expression, which is associated with tumorigenesis. In the non-canonical WNT/Ca^2+^ pathway, activation of CamKII by WNT5A induces the phosphorylation of SMRT (silencing mediator of retinoic acid and thyroid hormone receptor), resulting in increased promoter activity of a NOTCH-responsive gene (66).

### 3.4 KLFs in NOTCH signaling

The NOTCH signaling pathway is a juxtracrine signaling pathway that mediates cell fate decisions between neighbor cells. NOTCH consists of four paralogue genes (NOTCH 1-4) and five ligand genes (encoding Delta­like proteins DLL1, DLL3, and DLL4, Jagged 1 and Jagged 2). The NOTCH receptor is a transcription factor anchored at the membrane and is released following interaction with a cognate ligand (67). NOTCH signaling regulates various cellular processes, including proliferation, stem cell maintenance, and differentiation (68).

The NOTCH pathway plays a dual role in inhibiting and promoting tumors by regulating the tumor microenvironment (69). KLFs generally affect tumor growth by regulating the oncogenic properties of the NOTCH pathway (**Figure 1**). On the one hand, KLF2 and KLF9 inhibit oncogenesis, with the former restraining the hypoxia-inducible factor 1 (HIF-1α)/NOTCH-1 signaling pathway that mediates cell biological processes, including cell growth and apoptosis in colorectal cancer (70), and the latter inhibiting NOTCH-1 expression and downstream signaling in tumor-initiating stem cells by binding to the NOTCH-1 promoter (71). KLF16, on the other hand, is overexpressed in gastric cancer and promotes cancer cell growth and metastasis by activating the NOTCH pathway (72).

KLFs and the NOTCH signaling pathway may have a mutual regulatory relationship regarding tumorigenesis. KLF4, a vital regulator of the NOTCH pathway, promotes ineffective angiogenesis in tumors *via* the NOTCH pathway, leading to hypoxia and diminished tumor growth (73), and KLF4 also inhibits NOTCH-1 in combination with SP3 transcription (74). Interestingly, NOTCH-1 performs an inhibitory effect on KLF4 to regulate the proliferation and differentiation of cancer cells (75). An earlier study suggested that FoxD3 might bind to the NOTCH-1 response element in the KLF4 promoter to mediate the inhibition of KLF4 by NOTCH (76). However, this should be further investigated. Located upstream of the NOTCH pathway, Sin3A regulates the transcriptional capacity of the NOTCH receptor, which is necessary for the activation of NOTCH signaling (77, 78). KLF 9, 10, 11, 13, and 16, with a domain of combining Sin3 (11), may be involved in regulating the NOTCH pathway through interaction with Sin3A.

NOTCH activation and the disease outcome depend upon the crosstalk with other regulatory pathways, such as WNT signaling and NF-κB signaling. Loss of NOTCH-1 leads to activation of β-catenin, a key component of canonical WNT signaling, and increases the transcriptional activity of a β-catenin-responsive reporter construct, suggesting that NOTCH dampens beta-catenin-mediated responses to WNT (66). NOTCH intracellular domain (NICD) strongly interacts with NF-κB p65 in colorectal cancer, which resulted in the upregulation of B-cell lymphoma-extra large (Bcl-xL), leading to the inhibition of cell apoptosis (79).

### 3.5 KLFs in TGF-β signaling

The TGF-β family signaling pathway regulates gene transcription and influences cell proliferation, differentiation, and adhesion (80). The receptors for these ligands are serine/threonine kinases, and two distinct receptors are required for signaling, type I, and type II. In addition, many ligands require additional co-receptors. The signals from these receptors are predominantly transduced from the plasma membrane to the nucleus by the SMAD proteins, although several other non-SMAD signaling pathways can be activated in different contexts (81). This pathway is involved in tumor progression primarily through regulating EMT and immune cells in the tumor microenvironment (82, 83). Various KLFs regulate this pathway and enhance its role in cancer inhibition and promotion (**Figure 1**).

Among the KLFs, KLF4 may have the closest association with the TGF-β pathway. KLF4 is located upstream of the TGF-β pathway and inhibits the growth and migration of non-small cell lung cancer, cutaneous squamous cell carcinoma, and ovarian cancer cells by suppressing TGF-β-induced EMT (84–86). Further studies suggest that KLF4’s inhibition of cancer may be mediated by downregulating the TGF-β1-mediated extracellular regulated protein kinases (ERK)/JNK/NF-κB signaling pathway (22). However, the regulation of TGF-β by KLF4 is not unidirectional. Experiments demonstrated that miR-135a-5p is activated by TGF-β1 and downregulates KLF4 by interacting with its 3′-UTR (87), suggesting an antagonistic relationship between KLF4 and TGF-β. Under the paradoxical joint action of the two molecules, whether cancer cell proliferation is inhibited or promoted may depend on the expression level of E-cadherin, a factor with an essential role in EMT and regulated by both KLF4 and TGF-β1. Interestingly, KLF4 can also maintain cancer cells’ stemness and mesenchymal properties and promote cell proliferation in specific microenvironments and tumor types. Activated by KLF4, the TGF-β pathway can relieve KLF4 suppression by inhibiting miR-7-5p and jointly promote tumor cells to escape from cytotoxic T lymphocyte-mediated lysis (88, 89).

It has been investigated that KLF5 is acetylated by TGF-β in epidermal proliferation and involved in growth inhibition (2). Recent experiments prove that the acetylated KLF5 mediates the regulation of TGF-β on CXCR4 and Bcl-2, which promotes bone metastasis and docetaxel resistance of PC, revealing a pathway of TGF-β in oncogenesis (90, 91). KLF5 is also a mediator of the inhibitory effects of the TGF-β signaling on cancer that TGF-β/SMAD4 signal inhibits the transcription of KLF5 that, in turn, switches Sox4 from tumor promoter to suppressor (92). Additionally, RAS, a common oncogenic signal, interferes with the TGF-β-induced acetylation of KLF5 and interrupt the binding of the p300-KLF5-SMADs complex to gene promoters. This interference disables the regulatory functions of TGF-β to induce the p15 gene and repress the Myc gene through KLF5, further leading to the misexpression of oncogenes (93). These findings suggested that KLF5 mediated the crosstalk between TGF-β and Ras signaling, which is an important explanation for the shift of TGF-β from tumor suppressor to tumor promoter in addition to the 14-3-3ζ factor (94).

Several other KLFs are also involved in the regulation of the TGF-β pathway. KLF2 can inhibit the transcriptional activity of SMAD2/3 and SMAD4 in the TGF-β/SMAD pathway, improving TGF-β-induced target gene expression and inhibiting tumor growth and migration in HCC (95). KLF8 is activated by TGF-β1 through SMAD2 and promotes ovarian cancer progression (96). KLF16 is an oncogene in bladder cancer due to its inhibition of TGF-β receptor 3, which inhibits tumor proliferation by a non-canonical TGF-β pathway (97, 98). KLF17 is a crucial regulator of TGF-β signaling, which forms a positive feedback loop with SMAD3, and the expression of both is often positively correlated in cancer tissues. Mechanistically, TGF-β upregulates the expression of KLF17 through SMAD3, while KLF17 transcriptionally increases SMAD3 and enhances the binding of SMAD3 to the promoter of target genes. The deregulation of either KLF17 or SMAD3 would diminish the ability of TGF-β to inhibit cancer cells (99).

TGF-β signaling pathway plays a broad oncogenic/suppressive role in cancer by regulating other vital pathways in cancer development. TGF-β1 stimulation leads to a down-modulation of NF-κB/Rel activity by increasing IκBα expression, which induces the apoptosis of B cells (100). SMAD2/3 and NF-κB signaling support granulosa cell tumor (GCT) cell viability, and there is a positive feedback loop between NF-κB and SMAD3 signaling in late-stage GCT (101). TGF-β signaling is closely related to WNT signaling through cross points such as SMAD, axis inhibition, RhoA, and β-catenin. The genome-wide expression profile analysis of transgenic mice also showed that TGF-β/SMAD signaling pathway and WNT/β-catenin signaling pathway interact and regulate each other (102).

### 3.6 KLFs in MAPK signaling

Mitogen-activated protein kinase (MAPK) is a group of serine-threonine protein kinases that can be activated by different extracellular stimuli. The element composition of the MAPK signaling is a three-tier kinase pattern, including MAP kinase kinase kinase (MKKK), MAPK kinase kinase (MKK, including MEK and MKK), and MAPK (including ERK, p38 MAPK, and JNK). These kinases are activated sequentially and regulate various biological processes (103). The MAPK signaling pathway is often dysregulated in tumors and has received close attention because of its association with tumor energy metabolism and drug resistance (104). The ability of KLFs to regulate the MAPK pathway is increasingly being investigated (**Figure 1**).

KLF4 interacts with the MAPK signaling. Upregulated by MAPK signaling, KLF4 inhibits further activation of MAPK signaling, and the activation status of KLF4 antagonizes the phosphorylation process in MAPK signaling. Mechanically, the RAS/RAF/MEK/ERK signaling pathway upregulates the expression of E2F transcription factor 1 (E2F1), which binds to the promoter of KLF4, thereby increasing the expression of KLF4 at the protein and mRNA levels and reducing the apoptosis of cancer tissues (105). Other studies suggest that KLF4 inhibited the expression of phosphorylated JNK, ERK, and p38, which blocked the MAPK pathway signaling and thus inhibited the proliferation of tumor cells (106). Meanwhile, the phosphorylation process of ERK upregulates the ubiquitination and degradation process of KLF4 (107). However, another study suggests that KLF4 activates the p38 MAPK pathway in osteosarcoma, which is related to the context-dependent nature of KLF4 (108).

KLF5 is often an activator of MEK/ERK. KLF5 facilitates the activation of epidermal growth factor (EGF)-induced MEK/ERK signaling through transcriptional upregulation and activation of EGFR (109). Also, the activity of the KLF5 promoter is regulated by MEK and ERK, and the inhibitions of these two factors are detrimental to KLF5 expression (110), suggesting that KLF5 may be involved in the signal amplification process of the MAPK pathway. Notably, the function of KLF5 is also context-dependent. In cells during embryonic development, KLF5 binds to Spred1, a suppressor of ERK, and inhibits the level of phosphorylated ERK (111).

Other KLFs also have a regulatory relationship with the MAPK pathway. The expression of KLF15 is detrimental to ERK phosphorylation, thereby inhibiting the pro-proliferative effect of MAPK (112), while JNK and ERK have an inhibitory effect on KLF15 expression (113). KLF17 inhibits cancer cell invasion in lung adenocarcinoma by suppressing the phosphorylation of p38 MAPK and inhibiting the urokinase fibrinogen activator induced by p38 MAPK/SRC signaling pathway (114).

The MAPK pathway enhances the association of other signals with cancer. The NOTCH pathway promotes capillary-like sprout formation in cancerous tissues by activating neighboring endothelial cells, which requires MAPK signaling to induce the expression of Notch ligand Jagged1 (115). Animal experiments have demonstrated that the RAS/MAPK pathway and AKT/mTOR pathway act synergistically to increase tumor growth (116).

## 4 Implications of KLFs in tumor development

### 4.1 KLFs and the cell cycle

**Figure 2:**
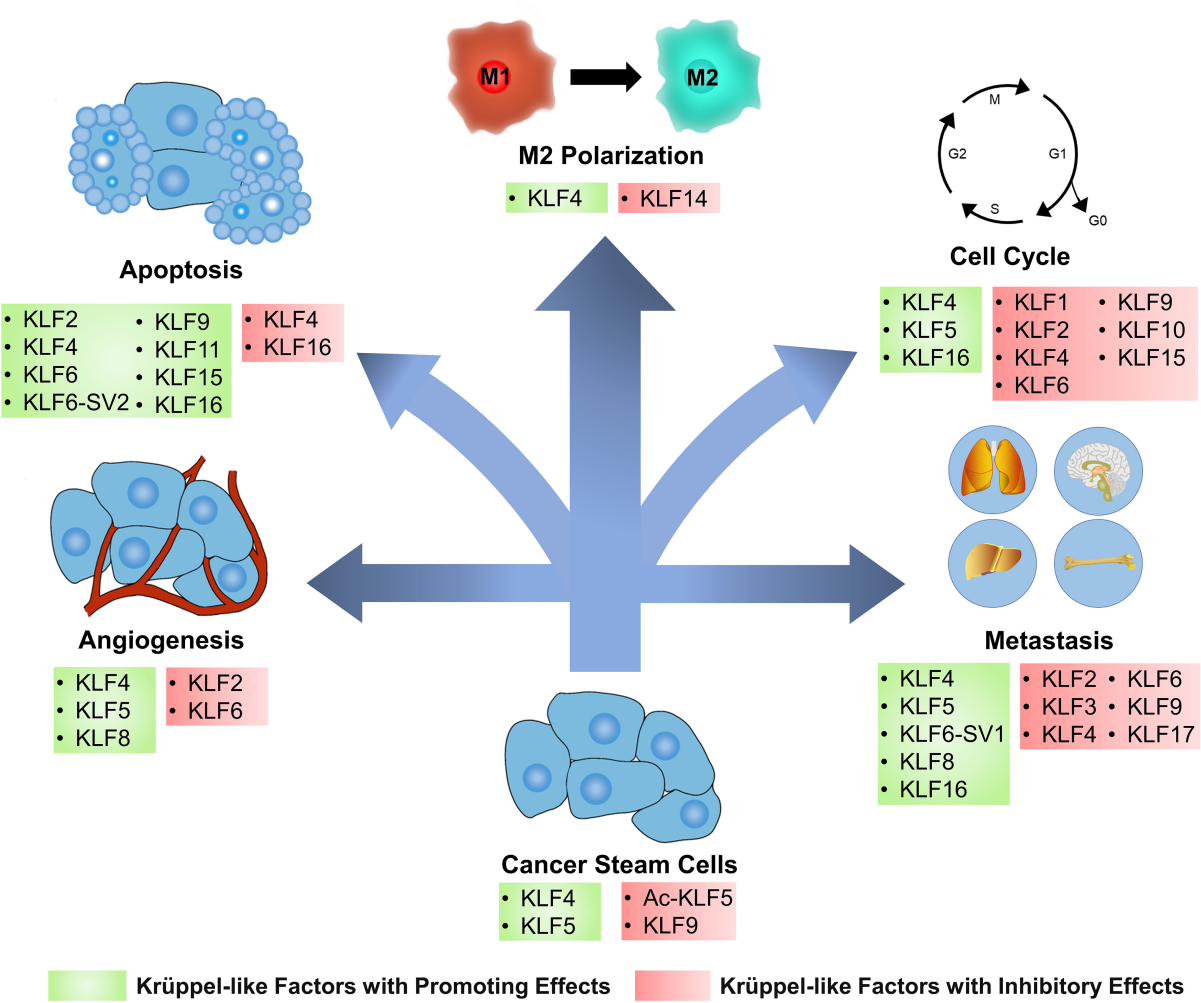
KLFs have diverse effects on cancer cells and tumor microenvironment. Based on the regulation of several signaling pathways and biomolecules, Krüppel-like factors (KLFs) regulate tumor progression by affecting several processes, including the cancer stemness, cell cycle, apoptosis, angiogenesis, metastasis, and cancer inflammation. Ac-KLF5, acetylated KLF5; M1, classically activated macrophage; M2, alternatively activated macrophage.

Orchestrated by a complex network of interactions between proteins, enzymes, cytokines, and cell cycle signaling pathways, cell cycle regulation is vital for cell proliferation, growth, and repair. KLFs can interact with cyclin-dependent kinases (CDKs), CDK inhibitors, and promoters of several transcription factors to participate in cell cycle regulation (2) (**Figure 2**).

Cell cycle checkpoints have cancericidal capacity due to their ability to monitor DNA damage, and defects often occur in various cancers. Depending on the type of damage and where in the cell cycle it occurs, different pathways are involved, such as the ATM-CHK2-p53-p21 pathway controlling the G1 checkpoint or ATR-CHK1-Wee1 pathway controlling the S and G2/M checkpoints (117). KLF2 can arrest the cell cycle at the G1 phase in BC and retinoblastoma cancer cells (118, 119). The up-regulation of p21 expression was also detected in the tissues, suggesting that KLF2 may arrest the cell cycle by assisting the expression of p21 (119). However, this regulation can be inhibited by Small nucleolar RNA host gene 6 (120). KLF4 is a cell cycle-blocking factor in tumor tissues such as PC and BC (121, 122). Mechanistically, KLF4 is enriched in the demethylated p21 promoter and increases its expression, thus blocking the cell cycle of cancer cells in the G1 phase (123). However, KLF4 can also act as an initiator of cancer cell proliferation in bladder cancer. Further studies have revealed that p21 is a molecular switch for the oncogenic/cancericidal role of KLF4. P21 promotes HDAC2 phosphorylation and acetylates KLF4 by interacting with CK2, the upstream kinase of HDAC2. The acetylated KLF4 can form positive feedback with p21, which further increases the proportion of acetylated KLF4 and the expression of the p21 gene (124). KLF6 also inhibits cancer cell proliferation through p21-mediated cyclin D1/CDK4 expression, but this pathway is regulated by miR-181a (125). However, not all KLFs act as blockers in the cell cycle of cancer cells. The over-expressed KLF5 directly inhibits p16 expression in pancreatic cancer to promote the G1/S phase transition of cancer cells (126). Interacting with the NF-κB p50 subunit, KLF5 also induces the expressions of positive cell cycle regulators cyclin B1 and CDK1/CDC2, which is critical to the development of the cell cycle (127). KLF5 also inhibits the transcription of cell cycle modulator p27kip1 in BC by regulating long non-coding RNA (LncRNA) RP1, resulting in oncogenesis (128). KLF16 overexpresses in oral squamous carcinoma and silencing this factor blocks cancer cells in the G0 and G1 phases (129). Besides, it also promotes cancer cell proliferation in gastric cancer by inhibiting the expression of the p21 gene (130).

KLFs are also associated with several transcription factors. GINS complex subunit 4 is a crucial factor required for the initiation and extension of DNA replication in the G1 to S phase in eukaryotes and an essential initiator in the development of colorectal cancer. KLF4 can bind to its promoter and downregulates its expression (131). KLF5 promotes the expression of E2F1, cyclin D1, and Rad51 in pancreatic cancer and assists in the G1/S phase progression of cancer cells (126). Meiotic nuclear divisions 1 (MND1) may competitively bind to KLF6, protecting E2F1 from KLF6-induced transcriptional repression and forming a positive feedback loop in the MND1-KLF6/E2F1 axis, thereby downregulating the inhibitory effect of KLF6 on cancer cells, which promotes cell cycle progression in cancer tissues (132).

Other KLFs can also regulate cancer cell proliferation by modulating the cell cycle. Silencing KLF1 blocks the cell cycle of gastric cancer cells in the G1 phase (57). KLF9 induces the arrest of cancer cells in the G2/M phase in PC and blocks the cell cycle in the S phase in lung adenocarcinoma, thereby inhibiting tumor growth (133, 134). KLF10 inhibits cancer by blocking cell cycle progression in multiple myeloma (56). KLF15 blocks the cell cycle in the G1 phase in BC (118).

### 4.2 KLFs in tumor cell apoptosis

Apoptosis, the most renowned form of programmed cell death, operates as a physiological mechanism that limits cell population expansion to maintain tissue homeostasis or remove potentially harmful cells, such as those that have sustained DNA damage (135). KLFs are involved in the control of tumor cell apoptosis by controlling various apoptotic genes and cell signaling pathways (**Figure 2**).

KLF4 typically promotes apoptosis in cancer cells but also exerts anti-apoptotic effects. KLF4 is upregulated in TNF-α-induced apoptosis and is essential in promoting the apoptotic process of BC cells (136). In gastric cancer, KLF4 promotes apoptosis in cancer cells by regulating inhibitors of apoptosis stimulating p53 protein (137). Furthermore, KLF4 is a monocyte chemotactic protein-induced protein 1 (MCPIP1)-dependent regulator in inflammation and innate immunity (138). During cellular autophagy/lysosomal degradation, MCPIP1 negatively regulates cancer cell apoptosis induced by death receptor 5 (DR5) transcription and DR5 activation (139), suggesting that KLF4 is involved in a novel pathway of apoptosis. However, in a nutrient-deprived environment, KLF4 protects cells from death by blocking the cell cycle and inhibiting apoptosis, and cancer hijacks the mitochondrial machinery to drive cell survival (140).

The role of KLF16 in cancer cell apoptosis has been a significant area of research in recent years. In glioblastoma, lncRNA RNCR3 can increase KLF16 expression by inhibiting miR-185-5p, and KLF16 acts as a direct target of this regulatory axis to induce apoptosis (141). However, KLF16 functions more as an inhibitor of apoptosis. In retinoblastoma, KLF16 suppresses the apoptotic gene Bcl2-like 15 and inhibits apoptosis by transcriptional upregulation of cellular retinoic acid-binding proteins-2 and activation of the integrin-β1/FAK/ERK pathway (142, 143). KLF16 expression was upregulated in oral squamous cell carcinoma, and interfering with KLF16 led to cell cycle arrest, inhibited tumor cell growth, and promoted cell apoptosis (129).

Several other KLFs are also associated with apoptosis in the cancer cell. KLF2 can activate mitochondria-mediated apoptosis in retinoblastoma (119). KLF6 induces apoptosis in non-small cell lung cancer cells by activating caspase-3, the most critical terminal splicing enzyme in apoptosis (144). An alternative splicing isoform of KLF6, KLF6-SV2, has been shown to induce apoptosis in colorectal cancer by increasing the expression of p21 and a Bcl-2 apoptotic protein Bax (145). KLF9 promotes apoptosis of cancer cells, especially androgen-dependent cells, in PC by severely suppressing the activation of AKT and its downstream targets and inhibiting the androgen receptor pathway (133, 146). KLF11 induces apoptosis by increasing intracellular reactive oxygen species levels (147). KLF2 and KLF15 also play a role in apoptosis in BC, but the exact pathway remains unknown (118).

### 4.3 KLFs in tumor metastasis

Most cancer-associated deaths are due to metastatic diseases rather than the primary tumor. Metastasis is a multistep process involving local invasion from the primary tumor, intravasation, survival in circulation, and extravasation, besides survival and proliferation in a target organ. The above process is summarized as the invasion-metastasis cascade, a relatively inefficient procedure since only a tiny proportion of cancer cells reach the final step of distant organ colonization (148). KLFs regulate tumor metastasis by altering the vascular permeability of tumor tissue and promoting or inhibiting EMT (**Figure 2**).

Enhancement of vascular permeability is indispensable for cancer metastasis. Vascular permeability is highly correlated with the extravasation of tumor cells because tumor cells must break through the endothelial cell barrier (149). KLF4 maintains the integrity of endothelial barrier function by promoting the promoter activity of tight junction-related proteins, including ZO-1, occludin, and Claudin-5 (150). These regulations are targeted by oncogenic exosomal miR-25-3p and miR-182-5p to induce vascular permeability (151, 152). KLF6 also enhances the promoter activity of tight junction-related proteins in endothelial cells, but it was inhibited by miR-181a and showed low expression in glioma (153).

Part of tumor metastasis from primary sites of malignancy to neighboring stromal tissue or distant organs entails EMT, which weakens the adhesion forces between differentiated epithelial cells so that tumor cells can achieve solitary or collective motility (154). KLFs indirectly affect EMT in cancer tissues by regulating MMP9, TWIST, and other factors or interacting with TGF-β, WNT, and other pathways. Overexpression of KLF3 abrogates fat mass and obesity-associated-induced osteosarcoma cell invasion (155) while silencing this factor promotes EMT and metastasis in lung cancer. KLF4 has a reciprocal regulatory relationship with TGF-β1 and thus affects EMT (84, 87). Colorectal cancer specimens with malignant features such as lymphatic metastasis showed low expression of KLF4 (156). Further studies revealed that KLF4 was negatively correlated with EMT indicators such as TWIST, β-catenin, claudin-1, N-cadherin, and vimentin, implying disrupted intestinal epithelial homeostasis, loss of cell polarity and intercellular junctions (157). KLF4 also indirectly modulates the actin cytoskeleton morphology *via* the activity of RhoA, thereby inhibiting the migration of colon cancer cells (158). However, KLF4 showed a facilitative effect on metastasis in advanced squamous cervical and esophageal cancers, which is regulated by miR-7-5p (51, 159). KLF6 inhibits metastasis in oral cancer cells by inhibiting MMP9 activity and impairing mesenchymal markers (160). In contrast to wild-type KLF6, KLF6-SV1 promoted the expression of TWIST1 and C-C motif ligand 2, which promoted the involvement of EMT in the metastasis of lung cancer cells (161). KLF9 inhibited BC metastasis through the upregulation of E-cadherin and downregulation of MMP9 expression (29, 162). Low expression of KLF17 mediated EMT by regulating the TGF-β pathway and EMT-related genes, and also increased cancer metastasis by upregulating inhibitor of DNA binding-1 (163).

Several KLFs regulate tumor metastasis by other pathways. KLF2 inhibits the migration of HCC cells by suppressing Hedgehog signaling. KLF6 inhibits ovarian cancer migration but is negatively regulated by multiple lncRNAs (164). KLF5 activates EMT in tumor tissues through PI3K/AKT/Snail signaling pathway (35), and high expression of KLF5 is associated with tumor metastasis and poor prognosis (165, 166). KLF8 induces BC metastasis by inducing the EPSTI1-VCP-NFκB signaling pathway (28). KLF16 is overexpressed in BC, and *in vitro* experiments showed that loss of KLF16 was detrimental to the migration of BC cells (167).

### 4.4 KLFs in the tumor angiogenesis

Tumor angiogenesis refers to the growth of new blood vessels within cancer tissue. Tumor blood vessels supply the tumor with oxygen and nutrients required for growth, remove waste products from tumor tissues, and provide a gateway for tumor metastasis (168). The function of KLFs in cancer angiogenesis varies by KLFs and tumor tissue (**Figure 2**).

KLF4 disturbs tumor angiogenesis by mediating the expression of members of NOTCH and the vascular endothelial growth factor (VEGF) signaling pathways. NOTCH signaling is central to the orchestration of sprouting angiogenesis. The sustained expression of KLF4 promotes the growth of hypo-perfused hypervascularity by regulating NOTCH, thus leading to reduced tumor growth (73). Mechanistically, KLF4 disables the activity of the essential NOTCH transcriptional activator RBP-J by interfering with the binding of co-activators NICD and MAML at intron 3 of the NOTCH ligand DLL4 (169). The VEGF pathway is another important signal that drives angiogenesis, and KLF4 can have contrasting regulatory functions depending on genetic background and co-regulatory factors. Experiments using retinal microvascular endothelial cells revealed that KLF4 increases VEGF expression, enhances the phosphorylation of VEGFR2 and activates downstream pERK1/2 and pAKT signaling to promote angiogenesis (150). In breast cancer cells, KLF4 recruits HDAC2 and HDAC3 to the VEGF promoter, inhibiting its angiogenic potential (170). Interestingly, VEGF also upregulates DLL4, and this activation is transmitted by AKT signaling, suggesting an assisting relationship between these two signaling pathways (169).

KLF5 promotes angiogenesis in bladder cancer by facilitating the transcription of VEGFA (171). Further studies revealed that HIF-1α mediates the regulation of VEGF by KLF5, and KLF5 can upregulate the expression of HIF-1α (172). However, a similar study of PTEN-deficient PC specimens reported a different result, suggesting that the regulation of HIF-1α and tumor angiogenesis by KLF5 is related to the presence or absence of the PTEN gene and the acetylation status of KLF5 (173). Another pro-angiogenic factor upregulated by KLF5 is platelet-derived growth factor alpha, but estrogens may suppress this KLF5-mediated transcription through estrogen receptors (174). In addition, KLF5 promotes the growth of tumor-associated fibroblasts, a type of cell thought to have an essential role in tumor angiogenesis (175).

In addition, KLF2 and KLF6 significantly reduced VEGFR2 expression to inhibit tumor angiogenesis (152, 176). KLF8 increased the activity of the VEGFA promoter and regulated HIF-1 alpha expression through activation of the PI3K/AKT signaling pathway, thereby inducing angiogenesis in cancer (177). These reports illustrate that the functions of KLFs in tumor angiogenesis are not irreplaceable.

### 4.5 KLFs in the tumor inflammation and immunology

The continuation of the inflammatory response plays a vital role in the initiation, promotion, malignant transformation, invasion, and metastasis of cancer (178). KLFs take part in the regulation of tumor-associated inflammation and inflammation in the tumor microenvironment (TME).

Chronic non-specific inflammation contributes to tumorigenesis. Ulcerative colitis may induce colon cancer, and KLF2 can inhibit the related inflammatory response by regulating the expression of IL-6, IL-8, IL-10, and TNF-α (179). The overexpression of KLF4 mediates chronic inflammation in the esophagus through the activation of NF-κB signaling, leading to epithelial hyperplasia followed by esophageal squamous cell carcinoma (23). In contrast, KLF5 protects tissues from T helper cell 17 (Th17)-mediated immune responses in intestinal epithelial cells, which facilitates protection against carcinogenesis due to excessive inflammation (180). KLF9 is a suppressor of liver inflammation that underlies its tumor-suppressive effects in the liver and other tissues (181).

Immune cells in TME play an essential role in tumor resistance and metastasis. The proliferation and survival of natural killer cells, an important immune cell being pursued in clinics to treat tumors, is supported by KLF2 (182). Regulated by multiple factors, tumor-associated macrophages (TAMs) often display anti-inflammatory and pro-tumor M2-like phenotypes, leading to poor immunotherapeutic outcomes (183). KLF4 and KLF14 are vital regulators of TAMs polarization. KLF4 mediates hedgehog-dependent TAM M2 polarization and immunosuppressive function (184), which promotes tumorigenesis. Additionally, KLF4 expression correlated with miR-34a-5p and IL-1 beta in a feed-forward loop, both implicated in immune regulation (185). By contrast, in BC, KLF14 reduced M2 macrophage polarization and related promoting factors of the tumor microenvironment (TGF-β, MMP9, and VEGF). Mechanistically, this is achieved by activating the transcription of suppressor of cytokine signaling-3, then blocking the activation of RhoA/Rock/Signal transducer and activator of transcription-3 (STAT3) signaling (186). STAT3 is a tumor promoter that helps tumor cells evade natural killer cell-mediated immune surveillance (187), and KLF5 is also associated with it. Downregulated KLF5 in prostate cancer activates STAT3, leading to immune escape and metastasis of tumors (188). However, KLF5 does not exclusively facilitate immune attacks on tumors. The protective effect of KLF5 on Th17-mediated immune response may lead to the immune escape of tumor tissues, as Th17 can recruit immune cells, secret the antitumor factor IFN-γ and is an essential mediator of adoptive immunotherapy (180, 189).

Consisting of CXC, CC, CX3C, and XC, the chemokine family can induce infiltration of immunosuppressive cells. The promoter region of the CXC ligand-8 (CXCL8) gene contains KLF4 binding sites, and the inactivation of KLF4 in gastric cancer tissues resulted in significant upregulation of the CXCL8 expression. By chemotactic bone marrow-derived suppressor cells, CXCL8 inhibits the activation of T cells and contributes to the immunosuppression of TME (190). Similarly, KLF5 mediates CXCL12 expression, creating an immunosuppressive microenvironment in tumor tissue and thus reducing the efficacy of immunotherapy (191).

### 4.6 KLFs and cancer stem cells

Stem cells are the longest-lived cells in many tissues, and their constant self-renewal provides the conditions for accumulating the genetic mutations required for cancer initiation. Cancer stem cells (CSCs) may be the source of all cancer cells and are responsible for tumor metastasis and recurrence after chemotherapy (192). KLFs regulates tissue differentiation by modulating stem cell activity in a physiological state, and associates with cancer stem cells (**Figure 2**).

KLF4 maintains stem cell self-renewal in homeostasis and cancer (193). In tumor tissues, KLF4 showed elevated expression levels in cell lines enriched with CSCs (194) and maintained self-renewal of CSCs by targeting dual-specificity tyrosine-(Y)-phosphorylation-regulated kinase 2 (DYRK2) and inducing telomerase expression. DYRK2 inhibits the survival and self-renewal of CSCs by activating p53 and degrading c-Myc, while KLF4 inhibits DYRK2 expression through binding to its promoter, resulting in cancer initiation and proliferation (195). High telomerase activity is necessary to maintain stem cells’ self-renewal and differentiation potential. By binding to poly (ADP-ribose) polymerase 1, KLF4 can localize to the promoter of telomerase reverse transcriptase and activate its expression (196). Based on the ability of KLF4 to activate telomerase, scholars can artificially induce somatic cells into pluripotent stem cells (197). The derived iPSC-NK cell therapies have shown promising safety and efficacy in tumor treatment (198).

Conversely, KLF9 inhibits tumor stem cells, suppressing the proliferation and metastasis of tumor tissue. Mechanistically, KLF9 strongly inhibits NOTCH-1 expression to modulate the stem-like properties negatively (199). By binding to the promoter of integrin α6, KLF9 highly suppresses the integrin signaling pathway, which is detrimental to the self-renewal of CSCs (200). However, some CSCs target KLF9 by upregulating miR-600, thus rescuing this repressive effect (201).

The acetylation level of KLF5 correlates with its ability to regulate embryonic stem cells and CSCs but shows distinct regulatory directions. In embryonic stem cells, acetylated KLF5 promotes self-renewal and inhibits cell differentiation (202). However, in tumor tissues, acetylated KLF5 was detrimental to the growth of CSCs-rich cell lines (203).

## 5 Applications of KLFs in precision medicine

### 5.1 KLFs as biomarkers in tumors

Despite significant progressions in cancer diagnosis and treatment strategies, cancer incidence and mortality rates remain high, causing great suffering and a considerable financial burden on patients and their families. Since early clinical signs are difficult to detect and many cancer patients are not diagnosed until the disease has progressed to an advanced stage, identifying valuable biomarkers is critical for cancer screening and prognosis. Numerous studies and analyses have discovered a strong link between abnormal expression of various KLFs and cancer prognosis.

Aberrant expression of several KLFs is strongly associated with tumorigenesis: KLF2 is a prognostic factor in HCC and BC (118, 204); KLF3 is low expressed in the peripheral blood of acute leukemia (205); KLF7 has been identified as an unfavorable prognostic marker in lung adenocarcinoma, gastric cancer, and high-grade plasma ovarian cancer (206, 207). KLF8 has a negatively associated expression level with miR-429 in osteosarcoma, and an elevated KLF8/miR-429 ratio suggests a high tumorigenic potential (208). The overexpression of KLF16 can be used to distinguish lung and gastric cancer from nontumor tissues (72, 209).

Abnormal expression of other KLFs may indicate the metastases of tumor tissue: The high KLF4 expression level in cervical cancer significantly indicates distant metastasis (159). KLF8 is significantly overexpressed in gastric cancer with liver metastases (210). The expression level of KLF16 suggests lymph node status and the possibility of distant metastases in gastric cancer tissue (72). Besides, KLF17 indicates metastasis in colorectal cancer (211).

Moreover, a few KLFs are associated with the effectiveness of tumor treatment and the probability of tumor recurrence: The high KLF4 expression level in cervical cancer is significantly related to resistance to radiation therapy and local recurrence (159). In prostate cancer, higher levels of KLF5 expression and lower levels of KLF13 expression suggest that patients can achieve better outcomes with immunotherapy and chemotherapy (212). However, upregulated KLF5 indicates the poor outcome of preoperative chemoradiation therapy for patients with rectal cancer, emphasizing the context-dependent nature of KLFs (213). Patients with squamous cell carcinoma of the head and neck with high expression of KLF6 are at higher risk of local recurrence after treatment (214). Additionally, the expression of KLF3, KLF5, KLF6, and KLF15 was highly correlated with the level of immune cell infiltration, suggesting that KLFs can be monitoring markers of immunotherapeutic efficacy and help in developing new immunotherapeutic agents by providing immunological information (215).

However, large-scale studies are still needed to confirm the possible clinical application of KLFs as biomarkers in cancer treatment and to explore the specific role played by KLFs in cancer progression.

### 5.2 KLFs as therapeutic targets in tumors

Owing to their essential roles in cancer progression, KLFs have the potential to be efficient therapeutic targets.

As KLFs are direct targets of some microRNAs and long non-coding RNAs, screening or synthesis of novel drugs targeting these ncRNAs may contribute to valuable therapeutic strategies. MiRNA dysregulation is a common feature of cancer, and due to the promiscuity of miRNA binding, this can result in a wide array of genes whose expression is altered. Some miRNAs promote cancer progression by inhibiting the tumor-suppressing KLFs, which could be attenuated by the overexpression of cancericidal KLFs (216). As for KLFs that promote cancer progression, some miRNAs become practical tools to suppress KLF-related cancers because they can directly target KLFs (217). LncRNAs are novel regulators for post-transcriptional gene expression and altered lncRNA function and expression are associated with tumorigenesis and cancer progression. Among lncRNAs, differentiation antagonizing non-protein coding RNA (DANCR) showed the most extensive regulation with KLFs. DANCR exhibits different biological functions in different tumor types: KLF12 mediates the promotive effect of DANCR in HCC (218), while KLF9 is the ultimate target of the cancer-inhibiting effect of DANCR in multiple myeloma (219). By upregulating anticancer KLFs or knocking down KLFs that mediate carcinogenesis, clinicians can mitigate the detrimental effects of lncRNA maladjustment on tumorigenesis.

KLFs can also be the targets for the standard treatments of tumors, such as chemotherapy and radiotherapy. On the one hand, KLFs mediate the treatment based on the regulation of oncogenic signaling pathways by conventional molecular drugs. Mesalazine can promote the sequestration of β-catenin to the plasma membrane, which is partially caused by the direct binding of KLF4 to β-catenin (220). Tetra methyl pyrazine inhibits the growth of Lewis lung carcinoma effectively with cooperration with cisplatin by reducing the expression of angiogenesis-promoting factor VEGF and increasing the expression of angiogenesis-inhibiting KLF4 (221). On the other hand, KLFs also play a critical role in response to radiotherapy. KLF5 is an upstream transcription factor that promotes the expression of miR-125b-5p, which hinders cell proliferation and strengthens radio sensitivity in BC (222). In lung cancer, KLF11 overexpresses after radiotherapy, which induces apoptosis and inhibits cell proliferation by increasing intracellular reactive oxygen levels (147).

Additionally, some KLFs and their aberrant expression contribute to the ability of tumor tissues to acquire drug resistance and develop metastasis, so modulation of their expression may help improve clinical efficacy. KLF4 induces the development of sorafenib resistance within HCC, and the inhibition of KLF4 expression with short hairpin RNA recovered the response of sorafenib-resisted HCC cell lines to sorafenib (223). The increased methylation level of CpG sites in the KLF4 promoter and decreased expression level of KLF4 in BC tissues lead to the resistance to paclitaxel, which has become the target of 3,3’-diindolylmethane in improving the effectiveness of cancer chemotherapy (224).

Finally, small molecule inhibitors are important tools in the treatment of advanced refractory cancers and recurrent or metastatic cancers (225). As the targets and functions of KLFs continue to be investigated, small molecule inhibitors of KLFs have also been developed. ML264, as a KLF5 inhibitor, significantly attenuated the migration ability and colonization of BC cells and osteosarcoma cells *in vitro* experiments, suggesting a potential clinical application (226, 227). Other small molecule inhibitors with similar effects to ML264 include SR18662, CID5951923, and SR15006, which have different inhibitory efficiencies against KLF5, but all have shown potential for clinical applications (60). Kenpaullone targets KLF4 and has been shown in animal studies to inhibit the proliferation and migration of cancer cells (228). Initially developed as a c-Myc inhibitor, APTO-253 has been found to induce KLF4 and is used as a KLF4 activator (229).

## 6 Conclusions and future perspectives

KLFs are recognized as critical regulators in many crucial biological processes, including cell proliferation, differentiation, survival, cell cycle, EMT, invasion, metastasis, specification, and maturation of cells, as well as organogenesis. This may depend on the following characteristics: Firstly, KLFs can transcriptionally regulate the expression of their target genes involved in this progress. Secondly, KLFs target crucial signaling pathways, such as PI3K/AKT, WNT, NOTCH, TGF-β, NF-κB, and MAPK. It may also crosstalk with other signaling pathways. Thirdly, it regulates cellular functions by cooperating with other regulators, such as SMAD3 and HDAC1/2.

Conversely, the dysregulation of KLFs function contributes to the occurrence of tumors. Anti-cancer KLFs are often negatively regulated during tumor progression, while the overexpression of oncogenic KLFs effects occurs in tumor tissues. Owing to its central role in tumor regulation, it has excellent potential as a therapeutic target for a few tumors. Multiple lncRNA and miRNA have been identified as upstream regulators of KLFs, which provides a viable pathway for targeting KLFs.

Although the role of each KLF in different tumor tissues is gradually being revealed, the compensatory and reciprocal regulation between KLFs remains a research gap. For example, KLF 2,6 and 8 play a similar or similar role in tumor angiogenesis. Studies in this field help further improve the efficacy of tumor therapies targeting KLFs, as blockade of the expression of compensable KLF may not completely prevent tumor growth and invasion. There is also a need to investigate the temporal and spatial differences in the expression of KLFs and their regulatory axes, which may provide necessary information and ideas to target KLFs for precision medicine.

Targeting and blocking the cancer-promoting KLFs in tumor tissues is another challenge. In addition to participating in tumor progression, KLFs also significantly contribute to many physiological processes, such as metabolism and cell differentiation. Interfering with KLFs expression to block tumor progression may adversely affect other human physiological functions. Confirming the safety of interfering with KLFs will further promote clinical translational research on interfering with KLFs.

Moreover, small interfering RNAs (siRNAs) and small molecule inhibitors often used in *in vitro* experiments to interfere with the expression of KLFs may become a new idea for targeting KLFs. A similar example is STP705, a siRNA drug used to interfere with TGF-β1 and COX-2 gene expression in squamous cell carcinoma in situ, which started its phase 2 clinical trial in August 2022. A growing number of studies have also confirmed the efficacy and safety of KLF inhibitors such as ML264, suggesting that KLFs are increasingly likely to be a new direction for oncogene-targeted therapy.

In conclusion, KLFs have essential functions in normal cellular processes and multiple cellular environments related to tumors, and there may be potential mutual regulatory relationships. By further elucidating these, we will better define the role of these vital transcriptional regulators as targets for diagnostic and therapeutic intervention.

## Author contributions

YZ and CY wrote and revised the manuscript. CY, XW, and CZ conceived the project and designed the review. DJ, DH, and LQ searched relevant research and participated in the manuscript discussion. SL and ZJ revised and finalized the manuscript. All authors contributed to the article and approved the submitted version.
