# Supplementary material for: Krüppel-like factors in tumors: Key regulators and therapeutic avenues
Source: Front Oncol. 2023 Jan 25;13:1080720. doi: 10.3389/fonc.2023.1080720 (PMC9905823; doi:10.3389/fonc.2023.1080720)
Supplement: Supplementary file 1 [file Table_1.docx]

**Supplementary file S1 | Cancers with alterations of KLFs**

| Tumor Type | Expression level | | References |
| --- | --- | --- | --- |
|  | **Decreased expression** | **Increased expression** |  |
| Bladder cancer | KLF4 | KLF5, KLF16 | (1-3) |
| Breast cancer | KLF2, KLF4, KLF6, KLF8-11, KLF14, KLF15, KLF17 | KLF5, KLF16 | (4-9) |
| Cervical cancer | KLF4(middle and low differentiation), KLF14 | KLF1, KLF4(high differentiation) | (10-12) |
| Clear cell carcinoma | KLF6 |  | (13) |
| Colorectal cancer | KLF2-6, KLF6-SV2, KLF8-10, KLF12-15, KLF17 | KLF1, KLF4(in spheroid cells), KLF5, KLF7, KLF16 | (1, 14-17) |
| Cutaneous squamous cell carcinoma | KLF4 |  | (18) |
| Endometrial cancer | KLF9 |  | (19) |
| Esophagus cancer | KLF4 | KLF5 | (1, 20) |
| Gastric cancer | KLF4, KLF6, KLF17 | KLF1, KLF5, KLF8, KLF16 | (1, 9, 21-26) |
| Glioma | KLF6, KLF9 |  | (27, 28) |
| Head and neck cancer | KLF6 | KLF5(in cell nuclei), KLF6(with higher risk of local recurrence) | (23, 29, 30) |
| Hemangioma |  | KLF7(during progressive phrase, and in infantile cancer cells) | (31) |
| Hepatocellular carcinoma | KLF2, KLF4, KLF6, KLF17(during progressive phrase) | KLF4(with sorafenib resistance), KLF5, KLF8 | (23, 32-37) |
| Leukemia | KLF3, KLF5 |  | (1, 38) |
| Lung cancer | KLF4-6, KLF17 | KLF7, KLF16 | (1, 39-42) |
| Lymphoma | KLF4 |  | (43) |
| Melanoma | KLF10 | KLF5 | (44, 45) |
| Myeloma | KLF9, KLF10 |  | (7, 46) |
| Oral squamous cell carcinoma | KLF6 | KLF16 | (23, 47) |
| Osteosarcoma |  | KLF3, KLF5, KLF8 | (48-50) |
| Ovarian cancer | KLF4 | KLF8 | (51, 52) |
| Pancreatic cancer | KLF9, KLF10(with higher risk of distant metastasis) | KLF5 | (53-55) |
| Prostate cancer | KLF3-13, KLF17 | KLF1, KLF15, KLF16 | (1, 56) |
| Retinoblastoma | KLF2 | KLF16 | (57, 58) |
| Thyroid cancer | KLF17 | KLF5 | (9, 59) |

Abbreviations: KLF, Krüppel-like factor

# Reference

1. Gao Y, Wu KJ, Chen YL, Zhou JC, Du C, Shi Q, et al. Beyond Proliferation: Klf5 Promotes Angiogenesis of Bladder Cancer through Directly Regulating Vegfa Transcription. *Oncotarget* (2015) 6(41):43791-805. doi: 10.18632/oncotarget.6101.

2. Chen XS, Wang P, Ou TW, Li J. Klf16 Downregulates the Expression of Tumor Suppressor Gene Tgfbr3 to Promote Bladder Cancer Proliferation and Migration. *Cancer Management and Research* (2022) 14:465-77. doi: 10.2147/cmar.S334521.

3. Ai X, Jia ZM, Liu SL, Wang JJ, Zhang X. Notch-1 Regulates Proliferation and Differentiation of Human Bladder Cancer Cell Lines by Inhibiting Expression of Kruppel-Like Factor 4. *Oncology Reports* (2014) 32(4):1459-64. doi: 10.3892/or.2014.3350.

4. Xiang FF, Zhu ZW, Zhang MZ, Wang J, Chen ZX, Li XX, et al. 3,3'-Diindolylmethane Enhances Paclitaxel Sensitivity by Suppressing Dnmt1-Mediated Klf4 Methylation in Breast Cancer. *Frontiers in Oncology* (2021) 11:627856. doi: 10.3389/fonc.2021.627856.

5. Bang S, Li JH, Zhang MQ, Cui RJ, Wu XW, Xin ZC, et al. The Clinical Relevance and Function of Kruppel-Like Factor 16 in Breast Cancer. *Cancer Management and Research* (2020) 12:6373-83. doi: 10.2147/cmar.S256490.

6. Zhu KY, Tian Y, Li YX, Meng QX, Ge J, Cao XC, et al. The Functions and Prognostic Value of Kruppel-Like Factors in Breast Cancer. *Cancer Cell International* (2022) 22(1):23. doi: 10.1186/s12935-022-02449-6.

7. Zhou MM, Chen JQ, Zhang H, Liu HL, Yao H, Wang XM, et al. Klf10 Inhibits Cell Growth by Regulating Pttg1 in Multiple Myeloma under the Regulation of Microrna-106b-5p. *International Journal of Biological Sciences* (2020) 16(12):2063-71. doi: 10.7150/ijbs.45999.

8. Chu J, Hu XC, Li CC, Li TY, Fan HW, Jiang GQ. Klf14 Alleviated Breast Cancer Invasion and M2 Macrophages Polarization through Modulating Socs3/Rhoa/Rock/Stat3 Signaling. *Cellular Signalling* (2022) 92:110242. doi: 10.1016/j.cellsig.2022.110242.

9. Zhou S, Tang X, Tang F. Krüppel-Like Factor 17, a Novel Tumor Suppressor: Its Low Expression Is Involved in Cancer Metastasis. *Tumor Biology* (2016) 37(2):1505-13. doi: 10.1007/s13277-015-4588-3.

10. Zhu BS, Liu QS, Han Q, Zeng BH, Chen JQ, Xiao QJ. Downregulation of Kruppel-Like Factor 1 Inhibits the Metastasis and Invasion of Cervical Cancer Cells. *Molecular Medicine Reports* (2018) 18(4):3932-40. doi: 10.3892/mmr.2018.9401.

11. Liu HX, Li N, Wei L, Zhou FX, Ma R, Xiao F, et al. High Expression of Kruppel-Like Factor 4 as a Predictor of Poor Prognosis for Cervical Cancer Patient Response to Radiotherapy. *Tumor Biology* (2017) 39(6):710225. doi: 10.1177/1010428317710225.

12. Lyu XR, Ding XC, Ye H, Guo R, Wu MH, Cao LL. Klf14 Targets Itgb1 to Inhibit the Progression of Cervical Cancer Via the Pi3k/Akt Signalling Pathway. *Discover Oncology* (2022) 13(1):30. doi: 10.1007/s12672-022-00494-1.

13. Yang F, Ma JJ, Tang QS, Zhang W, Fu Q, Sun JB, et al. Microrna-543 Promotes the Proliferation and Invasion of Clear Cell Renal Cell Carcinoma Cells by Targeting Kruppel-Like Factor 6. *Biomedicine & Pharmacotherapy* (2018) 97:616-23. doi: 10.1016/j.biopha.2017.10.136.

14. Jiang X, Shen TY, Lu HL, Shi CZ, Liu ZC, Qin HL, et al. Clinical Significance and Biological Role of Klf17 as a Tumour Suppressor in Colorectal Cancer. *Oncology Reports* (2019) 42(5):2117-29. doi: 10.3892/or.2019.7324.

15. Huang ZT, He HB, Qiu F, Qian HL. Expression and Prognosis Value of the Klf Family Members in Colorectal Cancer. *Journal of Oncology* (2022) 2022:6571272. doi: 10.1155/2022/6571272.

16. Zhang B, Guo DD, Zheng JY, Wu YA. Expression of Klf6-Sv2 in Colorectal Cancer and Its Impact on Proliferation and Apoptosis. *European Journal of Cancer Prevention* (2018) 27(1):20-6. doi: 10.1097/cej.0000000000000410.

17. Leng ZW, Tao KX, Xia QH, Tan J, Yue ZY, Chen JH, et al. Kruppel-Like Factor 4 Acts as an Oncogene in Colon Cancer Stem Cell-Enriched Spheroid Cells. *Plos One* (2013) 8(2):e56082. doi: 10.1371/journal.pone.0056082.

18. Li XM, Kim SJ, Hong DK, Jung KE, Choi CW, Seo YJ, et al. Klf4 Suppresses the Tumor Activity of Cutaneous Squamous Cell Carcinoma (Scc) Cells Via the Regulation of Smad Signaling and Sox2 Expression. *Biochemical and Biophysical Research Communications* (2019) 516(4):1110-5. doi: 10.1016/j.bbrc.2019.07.011.

19. Yan XF, Zhang HL, Ke JQ, Zhang YL, Dai CY, Zhu M, et al. Progesterone Receptor Inhibits the Proliferation and Invasion of Endometrial Cancer Cells by up Regulating Kruppel-Like Factor 9. *Translational Cancer Research* (2020) 9(4):2220-30. doi: 10.21037/tcr.2020.03.53.

20. Peng H, Chen D, Li D, Wei S. Mir-7-5p Targets Klf 4 Gene to Regulate the Proliferation and Migration of Esophageal Cancer Cells. *Tumor* (2021) 41(4):238-47. doi: 10.3781/j.issn.1000-7431.2021.11.325.

21. Yang TS, Chen M, Yang XH, Zhang XB, Zhang Z, Sun YY, et al. Down-Regulation of Klf5 in Cancer-Associated Fibroblasts Inhibit Gastric Cancer Cells Progression by Ccl5/Ccr5 Axis. *Cancer Biology & Therapy* (2017) 18(10):806-15. doi: 10.1080/15384047.2017.1373219.

22. Liu ZX, Wu X, Tian YY, Zhang WC, Qiao SY, Xu WT, et al. H. Pylori Infection Induces Cxcl8 Expression and Promotes Gastric Cancer Progress through Downregulating Klf4. *Molecular Carcinogenesis* (2021) 60(8):524-37. doi: 10.1002/mc.23309.

23. Hsu LS, Huang RH, Lai HW, Hsu HT, Sung WW, Hsieh MJ, et al. Klf6 Inhibited Oral Cancer Migration and Invasion Via Downregulation of Mesenchymal Markers and Inhibition of Mmp-9 Activities. *International Journal of Medical Sciences* (2017) 14(6):530-5. doi: 10.7150/ijms.19024.

24. Mao AW, Zhou X, Liu YX, Ding JB, Miao AY, Pan GF. Klf8 Is Associated with Poor Prognosis and Regulates Glycolysis by Targeting Glut4 in Gastric Cancer. *Journal of Cellular and Molecular Medicine* (2019) 23(8):5087-97. doi: 10.1111/jcmm.14378.

25. Ma P, Sun CQ, Wang YF, Pan YT, Chen QN, Liu WT, et al. Klf16 Promotes Proliferation in Gastric Cancer Cells Via Regulating P21 and Cdk4. *American Journal of Translational Research* (2017) 9(6):3027-36.

26. Li SY, Li Y, Tan BB, An ZJ. Krappel-Like Factor 1 Serves as a Facilitator in Gastric Cancer Progression Via Activating the Wnt/Beta-Catenin Pathway. *Acta Biochimica Polonica* (2021) 68(4):765-74. doi: 10.18388/abp.2020_5680.

27. Ying MY, Sang YY, Li YQ, Guerrero-Cazares H, Quinones-Hinojosa A, Vescovi AL, et al. Kruppel-Like Family of Transcription Factor 9, a Differentiation-Associated Transcription Factor, Suppresses Notch1 Signaling and Inhibits Glioblastoma-Initiating Stem Cells. *Stem Cells* (2011) 29(1):20-31. doi: 10.1002/stem.561.

28. Ma J, Yao YL, Wang P, Liu YH, Zhao LN, Li Z, et al. Mir-181a Regulates Blood-Tumor Barrier Permeability by Targeting Kruppel-Like Factor 6. *Journal of Cerebral Blood Flow and Metabolism* (2014) 34(11):1826-36. doi: 10.1038/jcbfm.2014.152.

29. Mao XH, Miao SS, He HJ, Miao SP, Pei R, Yang XG, et al. Kruppel-Like Factor 5: A Novel Biomarker for Lymph Node Metastasis and Recurrence in Supraglottic Squamous Cell Laryngeal Carcinoma. *Tumor Biology* (2014) 35(1):623-9. doi: 10.1007/s13277-013-1086-3.

30. Leon X, Venegas M, Pujol A, Bulboa C, Llansana A, Casasayas M, et al. Predictive Value of Transcriptional Expression of Kruppel-Like Factor-6 (Klf6) in Head and Neck Carcinoma Patients Treated with Radiotherapy. *Clinical & Translational Oncology* (2021) 23(12):2507-12. doi: 10.1007/s12094-021-02651-4.

31. Wu Y, Jin F, Huang H, Wang S. Suppression of Klf7 Gene Expression Inhibits Proliferation and Induces Apoptosis of Hemangioma Cells Via Nf-Kappa B Signaling Pathway. *Tropical Journal of Pharmaceutical Research* (2021) 20(7):1351-6. doi: 10.4314/tjpr.v20i7.5.

32. Pang LJ, Xu L, Yuan CW, Li XH, Zhang XY, Wang WJ, et al. Activation of Egfr-Klf4 Positive Feedback Loop Results in Acquired Resistance to Sorafenib in Hepatocellular Carcinoma. *Molecular Carcinogenesis* (2019) 58(11):2118-26. doi: 10.1002/mc.23102.

33. Yao SS, Tian C, Ding YC, Ye QW, Gao Y, Yang N, et al. Down-Reguation of Kruppel-Like Factor-4 by Microrna-135a-5p Promotes Proliferation and Metastasis in Hepatocellular Carcinoma by Transforming Growth Factor-Beta 1. *Oncotarget* (2016) 7(27):42566-78. doi: 10.18632/oncotarget.9934.

34. Ali A, Zhang P, Liangfang Y, Wenshe S, Wang H, Lin X, et al. Klf17 Empowers Tgf-Beta/Smad Signaling by Targeting Smad3-Dependent Pathway to Suppress Tumor Growth and Metastasis During Cancer Progression. *Cell Death & Disease* (2015) 6:e1681. doi: 10.1038/cddis.2015.48.

35. Lin JB, Tan HF, Nie YJ, Wu DW, Zheng WJ, Lin WS, et al. Kruppel-Like Factor 2 Inhibits Hepatocarcinogenesis through Negative Regulation of the Hedgehog Pathway. *Cancer Science* (2019) 110(4):1220-31. doi: 10.1111/cas.13961.

36. Shen YN, He HG, Shi Y, Cao J, Yuan JY, Wang ZC, et al. Kruppel-Like Factor 8 Promotes Cancer Stem Cell-Like Traits in Hepatocellular Carcinoma through Wnt/Beta-Catenin Signaling. *Molecular Carcinogenesis* (2017) 56(2):751-60. doi: 10.1002/mc.22532.

37. An TT, Dong TX, Zhou HX, Chen YD, Zhang JW, Zhang Y, et al. The Transcription Factor Kruppel-Like Factor 5 Promotes Cell Growth and Metastasis Via Activating Pi3k/Akt/Snail Signaling in Hepatocellular Carcinoma. *Biochemical and Biophysical Research Communications* (2019) 508(1):159-68. doi: 10.1016/j.bbrc.2018.11.084.

38. Yan M, Liu HH, Xu JH, Cen XN, Wang Q, Xu WL, et al. Expression of Human Kruppel-Like Factor 3 in Peripheral Blood as a Promising Biomarker for Acute Leukemia. *Cancer Medicine* (2020) 9(8):2803-11. doi: 10.1002/cam4.2911.

39. Hu WX, Jia YL, Xiao XS, Lv KZ, Chen YX, Wang LB, et al. Klf4 Downregulates Htert Expression and Telomerase Activity to Inhibit Lung Carcinoma Growth. *Oncotarget* (2016) 7(33):52870-87. doi: 10.18632/oncotarget.9141.

40. Jiao XD, Gao WN, Ren HX, Wu YN, Li TZ, Li SJ, et al. Kruppel Like Factor 16 Promotes Lung Adenocarcinoma Progression by Upregulating Lamin B2. *Bioengineered* (2022) 13(4):9482-94. doi: 10.1080/21655979.2022.2060780.

41. Zeng B, Lin J, Cai X, Che L, Zeng W, Liu S. Kruppel-Like Factor 6 Downregulation Is Connected with a Poor Prognosis and Tumor Growth in Non-Small-Cell Lung Cancer. *Computational and mathematical methods in medicine* (2022) 2022:1-11. doi: 10.1155/2022/3193553.

42. Cai XD, Che L, Lin JX, Huang SA, Li JO, Liu XY, et al. Kruppel-Like Factor 17 Inhibits Urokinase Plasminogen Activator Gene Expression to Suppress Cell Invasion through the Src/P38/Mapk Signaling Pathway in Human Lung Adenocarcionma. *Oncotarget* (2017) 8(24):38743-54. doi: 10.18632/oncotarget.17020.

43. Li XY, Geng LY, Zhou XX, Wei N, Fang XS, Li Y, et al. Kruppel-Like Factor 4 Contributes to the Pathogenesis of Mantle Cell Lymphoma. *Leukemia & Lymphoma* (2017) 58(10):2460-9. doi: 10.1080/10428194.2017.1292354.

44. Jia XM, Chen HW, Ren Y, Dejizhuoga, Gesangyuzhen, Gao NN, et al. Bap1 Antagonizes Wwp1-Mediated Transcription Factor Klf5 Ubiquitination and Inhibits Autophagy to Promote Melanoma Progression. *Experimental Cell Research* (2021) 402(1):112506. doi: 10.1016/j.yexcr.2021.112506.

45. Zhao ZR, Zhan YC, Jing L, Zhai HL. Klf10 Upregulates Acsm3 Via the Pi3k/Akt Signaling Pathway to Inhibit the Malignant Progression of Melanoma. *Oncology Letters* (2022) 23(6):175. doi: 10.3892/ol.2022.13295.

46. Wu LL, Xia L, Jiang HM, Hu Y, Li LL, Xu L, et al. Long Non-Coding Rna Dancr Represses the Viability, Migration and Invasion of Multiple Myeloma Cells by Sponging Mir-135b-5p to Target Klf9. *Molecular Medicine Reports* (2021) 24(3):649. doi: 10.3892/mmr.2021.12288.

47. Yang L, Shi YL, Ma Y, Ren WW, Pang GM, Liu J. Silencing Klf16 Inhibits Oral Squamous Cell Carcinoma Cell Proliferation by Arresting the Cell Cycle and Inducing Apoptosis. *Apmis* (2022) 130(1):43-52. doi: 10.1111/apm.13194.

48. Shan HJ, Gu WX, Duan G, Chen HL. Fat Mass and Obesity Associated (Fto)-Mediated N6-Methyladenosine Modification of Kruppel-Like Factor 3 (Klf3) Promotes Osteosarcoma Progression. *Bioengineered* (2022) 13(4):8038-50. doi: 10.1080/21655979.2022.2051785.

49. Zhang L, Yang P, Liu Q, Wang J, Yan F, Duan L, et al. Klf8 Promotes Cancer Stem Cell-Like Phenotypes in Osteosarcoma through Mir-429-Sox2 Signaling. *Neoplasma* (2020) 67(3):519-27. doi: 10.4149/neo_2020_190711N624.

50. Huang H, Han Y, Chen ZJ, Pan X, Yuan PT, Zhao XD, et al. Ml264 Inhibits Osteosarcoma Growth and Metastasis Via Inhibition of Jak2/Stat3 and Wnt/Beta-Catenin Signalling Pathways. *Journal of Cellular and Molecular Medicine* (2020) 24(10):5652-64. doi: 10.1111/jcmm.15226.

51. Chen ZX, Wang YN, Liu W, Zhao GN, Lee S, Balogh A, et al. Doxycycline Inducible Kruppel-Like Factor 4 Lentiviral Vector Mediates Mesenchymal to Epithelial Transition in Ovarian Cancer Cells. *Plos One* (2014) 9(8):e105331. doi: 10.1371/journal.pone.0105331.

52. Cherukunnath A, Davargaon RS, Ashraf R, Kamdar U, Srivastava AK, Tripathi PP, et al. Klf8 Is Activated by Tgf-Beta 1 Via Smad2 and Contributes to Ovarian Cancer Progression. *Journal of Cellular Biochemistry* (2022) 123(5):921-34. doi: 10.1002/jcb.30235.

53. Zhong ZW, Zhou F, Wang D, Wu MM, Zhou WM, Zou YQ, et al. Expression of Klf9 in Pancreatic Cancer and Its Effects on the Invasion, Migration, Apoptosis, Cell Cycle Distribution, and Proliferation of Pancreatic Cancer Cell Lines. *Oncology Reports* (2018) 40(6):3852-60. doi: 10.3892/or.2018.6760.

54. Li YL, Kong R, Chen HZ, Zhao ZJ, Li L, Li JT, et al. Overexpression of Klf5 Is Associated with Poor Survival and G1/S Progression in Pancreatic Cancer. *Aging-Us* (2019) 11(14):5035-57. doi: 10.18632/aging.102096.

55. Tsai YC, Chen SL, Peng SL, Tsai YL, Chang ZM, Chang VHS, et al. Upregulating Sirtuin 6 Ameliorates Glycolysis, Emt and Distant Metastasis of Pancreatic Adenocarcinoma with Kruppel-Like Factor 10 Deficiency. *Experimental and Molecular Medicine* (2021) 53(10):1623-35. doi: 10.1038/s12276-021-00687-8.

56. Meng JL, Lu XF, Zhou YJ, Zhang M, Gao L, Gao SL, et al. Characterization of the Prognostic Values and Response to Immunotherapy/Chemotherapy of Kruppel-Like Factors in Prostate Cancer. *Journal of Cellular and Molecular Medicine* (2020) 24(10):5797-810. doi: 10.1111/jcmm.15242.

57. Zhang JX, Yan XJ, Wu S, Liu Q, Ma JM. Klf16 Overexpression Deleteriously Affects the Proliferation and Migration of Retinoblastoma by Transcriptionally Repressing Bcl2l15. *Biochemical and Biophysical Research Communications* (2020) 529(4):977-83. doi: 10.1016/j.bbrc.2020.06.027.

58. Wu ND, Chen SL, Luo Q, Jiang ZH, Wang X, Li Y, et al. Kruppel-Like Factor 2 Acts as a Tumor Suppressor in Human Retinoblastoma. *Experimental Eye Research* (2022) 216:108955. doi: 10.1016/j.exer.2022.108955.

59. Ma YH, Wang QZ, Liu F, Ma XJ, Wu LN, Guo F, et al. Klf5 Promotes the Tumorigenesis and Metastatic Potential of Thyroid Cancer Cells through the Nf-Kappa B Signaling Pathway. *Oncology Reports* (2018) 40(5):2608-18. doi: 10.3892/or.2018.6687.
